# Tunable-Diameter Nanoscrolls from Janus WSSe/WSe_2_ Heterostructures

**DOI:** 10.1021/acsnano.5c10877

**Published:** 2025-09-23

**Authors:** Masahiko Kaneda, Wenjin Zhang, Dingkun Bi, Tianyishan Sun, Hiroto Ogura, Takahiko Endo, Yuta Takahashi, Shun Fujii, Toshiaki Kato, Yasumitsu Miyata

**Affiliations:** 1 Research Center for Materials Nanoarchitectonics (MANA), National Institute for Materials Science (NIMS), Tsukuba 305-0044, Japan; 2 Department of Physics, 12944 Tokyo Metropolitan University, Hachioji 192-0397, Japan; 3 Graduate School of Engineering, 13101 Tohoku University, Sendai 980-8579, Japan; 4 Advanced Institute for Materials Research (AIMR), 13101 Tohoku University, Sendai 980-8577, Japan; 5 Department of Physics, Faculty of Science and Technology, 74013 Keio University, Yokohama 223-8522, Japan

**Keywords:** transition metal dichalcogenides, Janus WSSe, nanoscrolls, plasma treatment, bending rigidity, second-harmonic generations

## Abstract

Janus transition metal dichalcogenide (TMD) nanoscrolls have recently emerged as promising nanostructures for studying curvature- and chirality-dependent physical phenomena. However, systematic strategies to fabricate multilayer Janus TMD nanoscrolls with controlled diameters and to probe their structure-dependent optical behaviors are still lacking. Expanding on the previous finding that Janus TMD monolayers—with intrinsic asymmetry and built-in strain—spontaneously form nanoscrolls, we now demonstrate diameter-tunable nanoscrolls derived from Janus WSSe/WSe_2_ heterostructures. The incorporation of a Janus monolayer facilitates the scrolling of heterostructures and enables continuous tuning of nanoscroll diameters across a broad range—from ∼10 nm to ∼1 μm. The resulting structures exhibit uniform crystallinity and composition, as confirmed by scanning transmission electron microscopy. Optical characterizations reveal anisotropic Raman responses and strain-induced modulation of second-harmonic generation (SHG). These results indicate that Janus-based nanoscrolls provide a versatile platform for investigating structure–property relationships and developing rolled TMD systems for advanced photonic and optoelectronic applications.

## Graphical abstract

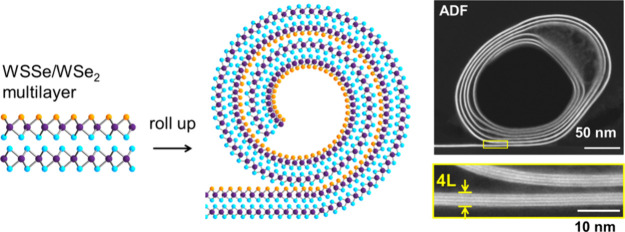

Tubular structures of transition metal dichalcogenide (TMD) exhibit unique structural and electronic properties that have inspired applications ranging from transistors and optoelectronics to thermoelectrics and sensing. Since the first synthesis of multiwalled TMD nanotubes in 1992,^1^ they have found applications in diverse fields, including polymer composites,^2^ catalysts,^3,4^ sensors,^5^ field-effect transistors,^6,7^ optoelectronic devices,^8,9^ memory devices,^10,11^ and thermoelectric devices.^12^ Theoretical studies predict that their electronic structure depends sensitively on diameter, number of layers, and chirality,^13–15^ motivating efforts to control these parameters experimentally. Small-diameter TMD nanotubes show pronounced curvature effects, including red-shifted photoluminescence due to bandgap reduction.^16^ In contrast, larger tubes (∼100 nm) support strong light-matter coupling, such as exciton polaritons,^17,18^ and optical microcavity resonators.^19^ Chiral TMD nanotubes have also demonstrated nonreciprocal superconductivity^20^ and giant bulk photovoltaic effects,^21^ likely due to their chiral structure. Despite these advances, available samples are largely limited to multiwalled nanotubes with mixed crystal orientations, which complicates structure–property analysis and functional control. Achieving structurally uniform and tunable TMD nanotubes remains a key challenge for advancing their applications.

To overcome these limitations, nanoscrolls, formed by rolling TMD sheets, offer an alternative strategy to construct tubular structures with well-defined crystal orientation. In contrast to conventional nanotubes, nanoscrolls derived from single-crystal TMDs maintain coherent stacking, enabling systematic exploration of structure–property relationships. Since their first fabrication in 2016,^22^ TMD nanoscrolls have been investigated for use in photodetectors,^23^ gas sensors,^24,25^ and catalysts.^26,27^ Two main fabrication approaches have been reported: strain-induced scrolling and solution-assisted scrolling. The former relies on lattice strain generated by sulfur vacancy defects,^22,28^ but typically compromises crystallinity, limiting the quality of the resulting nanoscrolls. The latter uses solvents introduced between the TMD sheets and their substrates, where surface tension drives the scrolling.^23,29–36^ However, conventional TMDs favor flat structures to relieve strain between chalcogen and metal atoms, often resulting in ribbon-like morphologies.

To address the structural instability of conventional TMD sheets, narrow cylindrical nanoscrolls have been fabricated using Janus monolayers composed of transition metals sandwiched between two different chalcogen atoms. Sayyad et al. first reported Janus TMD nanoscrolls formed by spontaneous rolling of monolayer sheets.^37^ More recently, we demonstrated that the intrinsic asymmetry of Janus TMDs enables the formation of stable nanoscrolls with inner diameters as small as 5 nm, as confirmed by electron microscopy and ab initio calculations.^38,39^ However, these previous studies have focused exclusively on monolayer sheets. Extending this approach to multilayer TMDs by leveraging the internal strain in Janus sheets offers a viable route to fabricating nanoscrolls with tunable diameters, as the increased bending rigidity of thicker TMD stacks naturally leads to larger scroll diameters.

Here, we report the diameter control of Janus TMD nanoscrolls and their structure-dependent properties. By varying the layer number of Janus TMD sheets, we fabricated nanoscrolls with a wide range of diameters. Janus WSSe sheets were prepared via plasma-assisted surface atom substitution and then rolled into nanoscrolls through a solution-based process. The resulting scrolls were characterized by atomic force microscopy (AFM) and high-angle annular dark-field scanning transmission electron microscopy (HAADF-STEM). The photostability of Janus WSSe nanoscrolls was assessed using photoluminescence (PL) and Raman spectroscopy. Their anisotropic optical response was investigated through polarized angle-resolved Raman measurements. Nonlinear optical properties induced by axial strain of nanoscrolls were characterized by polarization-resolved second-harmonic generation (SHG).

## Results and Discussion

Figure 1a-d illustrates the fabrication process of Janus WSSe based nanoscrolls. Janus WSSe is prepared by plasma-assisted atom substitution at room temperature (Figure 1a).^40^ In this process, the Se atoms on the top surface of the WSe_2_ are selectively replaced by S atoms to form a Janus WSSe structure (Figure 1b). For multilayer samples, only the topmost Se layer is substituted, resulting in the formation of a WSSe/WSe_2_ heterostructure (Figure 1b). Figure 1c,d shows the structural models of nanoscrolls with different compositions. Nanoscrolls composed entirely of WSSe are formed by rolling Janus monolayers (Figure 1c), while scrolls composed of both WSSe and WSe_2_ layers are obtained by rolling bilayer or thicker heterostructures (Figure 1d). Thus, by tuning the number of layers, nanoscrolls with different morphologies and compositions can be systematically designed.

**Figure 1:**
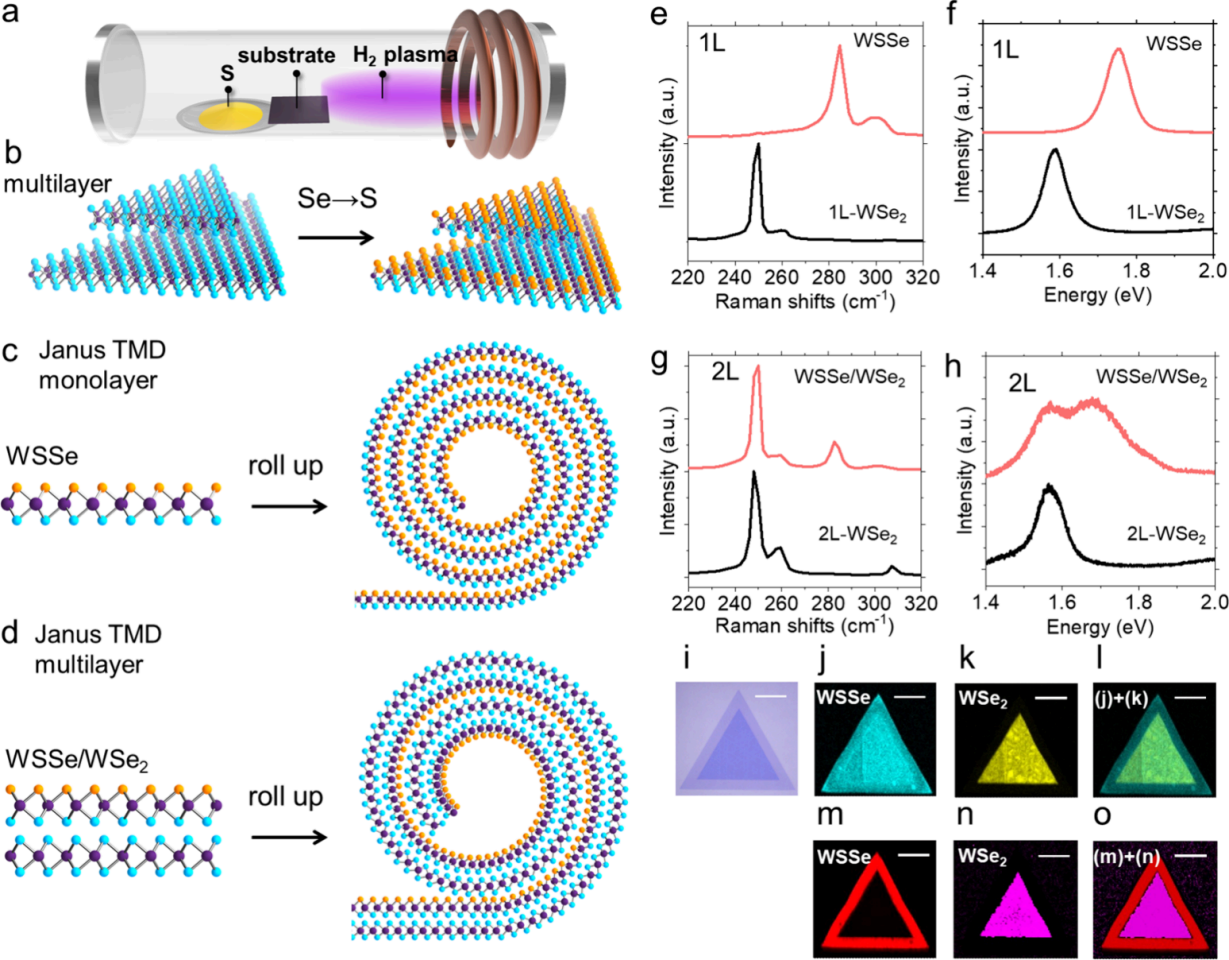
**Fabrication and characterization of Janus WSSe/WSe_2_ heterostructure.** (a) Schematic illustration of the hydrogen plasma-assisted sulfurization process. Structural models of (b) WSe_2_ bilayer and WSSe/WSe_2_ heterobilayer, (c) Janus WSSe monolayer and the corresponding monolayer-based nanoscroll, and (d) WSSe/WSe_2_ heterobilayer and the corresponding nanoscroll. (e) Raman and (f) PL spectra of CVD-grown WSe_2_ monolayer and WSSe monolayer. (g) Raman and (h) PL spectra of CVD-grown WSe_2_ bilayer and WSSe/WSe_2_ heterobilayer. (i) Optical microscopy image of WSSe/WSe_2_ after plasma treatment. Raman intensity maps of (j) A_1_ mode of WSSe at 288 cm^–1^, (k) A_1_′ mode of WSe_2_ at 250 cm^–1^, and (l) their superposition (WSSe+WSe_2_). PL intensity maps of (m) the WSSe peak at 1.7 eV, (n) the WSe_2_ peak at 1.6 eV, and (o) their combined intensity. Scale bars in (i–o), 20 μm.

The fabrication process starts with the growth of a single-crystalline WSe_2_ on a SiO_2_/Si substrate by chemical vapor deposition (CVD). Figure S1 shows the optical microscopy images after the atom substitution process. The number of layers was identified by optical contrast, which revealed a bilayer region stacked over triangular monolayer domains. The formation of Janus WSSe was confirmed by Raman and PL spectroscopy. Figure 1 e-h shows the Raman and PL spectra of the samples before and after the substitution process. In the monolayer region, the spectra exhibit a peak at 288 cm^–1^ and 1.70 eV (Figure 1e,f), corresponding to the A_1_ Raman mode and A exciton of Janus WSSe, respectively, consistent with previous reports.^40,41^ In the bilayer region, the Raman spectra exhibit peaks at 250 cm^–^^1^ and 288 cm^–^^1^, while the PL spectra show peaks at 1.6 and 1.7 eV (Figure 1g,h). These results indicate that atomic substitution has occurred only in the topmost layer of WSe_2_, forming a WSSe/WSe_2_ heterobilayer, as reported previously.^40,42^ Raman and PL intensity maps confirm uniform substitution of surface Se atoms with S atoms throughout the triangular grains (Figure 1i-o). Finally, the substrates were spin-coated with the PMMA/chloroform solution to prepare the nanoscrolls and rinsed with acetone. As reported in our previous work,^38^ this solution-based treatment serves as a trigger for the rolling of TMD sheets (Figure S1).

To characterize the nanoscroll structure, AFM was first used to study the surface topography and to estimate the number of layers in the precursor TMD sheets. Figure 2a–c shows topography images of nanoscrolls formed from WSSe monolayers, WSSe/WSe_2_ bilayers, and WSSe/3L-WSe_2_ four-layer sheets after solution treatments. In most regions, the sheets roll up from multiple directions and typically terminate upon encountering other nanoscrolls. As a result, individual nanoscrolls exhibit random orientations, suggesting no preferential rolling direction in TMD sheets. We note that alignment control of monolayer nanoscrolls has been demonstrated by patterning of MoS_2_ films with a focused ion beam.^29^ However, the alignment of multilayer nanoscrolls remains technically challenging due to the difficulty of forming uniform films over large areas. Patterning for Janus conversion is also considered an effective approach for orientation control.^43^ Figure 2d-f shows the corresponding height profiles extracted along the white lines in Figure 2a–c. The nanoscroll height exhibits variation within each sample, suggesting differences in interlayer spacing, layer number, and scroll diameter.

**Figure 2:**
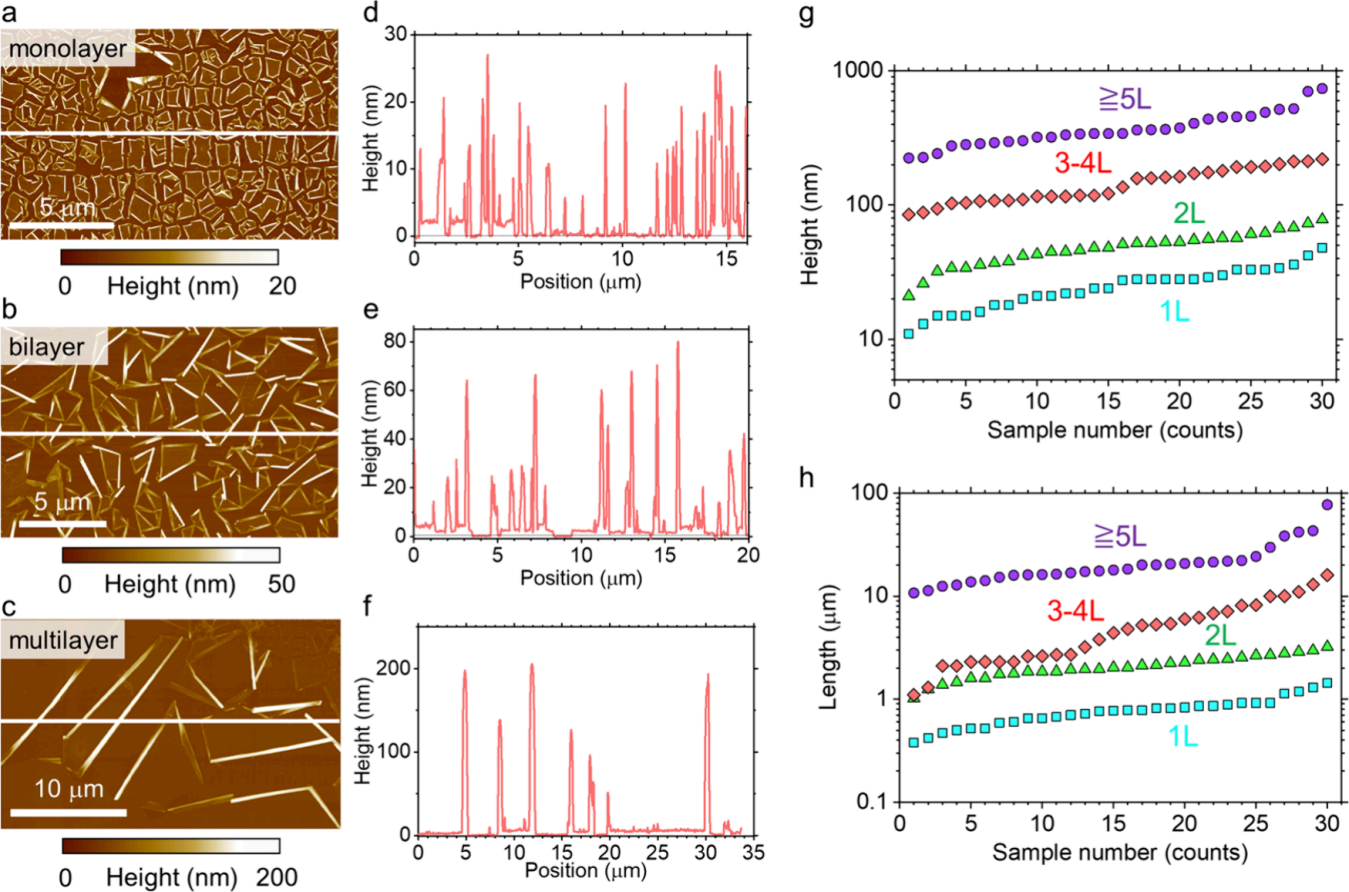
Topographic characterization of Janus WSSe-based nanoscrolls. AFM images of nanoscrolls formed from (a) monolayer, (b) bilayer, and (c) multilayer sheets after solution treatment. (d–f) Height profiles along the white lines shown in (a–c), respectively. (g) Height and (h) length distributions of nanoscrolls formed from sheets with different initial thicknesses: monolayer (1L, cyan squares), bilayer (2L, green triangles), trilayer–four-layer (3–4 L, red diamonds), and multilayer (≥5L, purple circles).

Figure 2g summarizes the height distributions for scrolls formed from monolayer, bilayer, trilayer, four-layer, and multilayer (>5 layers) sheets. The scroll height ranges from 11 to 48 nm for monolayers, from 21 to 78 nm for bilayers, from 85 to 140 nm for trilayers, from 160 to 220 nm for four-layers, and from 220 to 730 nm for thicker multilayers. These results reveal a clear correlation between nanoscroll diameter and the number of layers in the precursor sheet. This trend arises from the relationship between layer number and bending stiffness. In Janus TMDs, scrolling is driven by the relaxation of intrinsic strain associated with the asymmetric structure. Monolayer WSSe readily forms narrow nanoscrolls, as the strain relaxation provides a significant energy gain. In contrast, when WSSe is stacked on top of WSe_2_—forming bilayers or thicker heterostructures—the added layers increase the overall bending stiffness and reduce the energy gain associated with narrow curvature. Consequently, the critical curvature required to stabilize a scroll shifts toward larger values with increasing layer number. These findings demonstrate that the nanoscroll diameter can be effectively tuned by controlling the number of layers in the starting sheet.

The dependence of the scroll diameter on the layer number can be qualitatively understood by considering the bending of an elastic plate. Figure S2 shows the calculated strain energy density versus diameter, as well as the most stable diameter for a monolayer (*N* = 1) and heterostructures (*N* = 2∼4). Here, the strain energy was evaluated for the two limiting cases: a perfectly bonded multilayer and independently sliding layers as previously discussed.^44^ Details are provided in the Supporting Information. In both cases, the strain energy density increases rapidly below *d*_eff_, which limits the minimum inner diameter formed in the scroll (Figure S2a,b). Figure S2c shows that the experimental inner diameters lie between these two limits. This trend can be attributed to partial interlayer sliding in nanoscrolls, as reported in bending experiments on multilayer MoS_2_.^44^ A more precise statistical evaluation of the inner diameter and a theoretical assessment of the bending rigidity are left for future work.

Similarly, Figure 2h presents the length distributions of nanoscrolls formed from monolayer, bilayer, trilayer, four-layer, and multilayer (>5 layers) sheets. Measured scroll lengths range from 0.4 to 1.4 μm for monolayers, from 1.0 to 3.2 μm for bilayers, from 1.1 to 8.2 μm for trilayers, from 2.1 to 16.1 μm for four-layers, and from 10.7 to 77.4 μm for thicker multilayers. Again, a clear correlation is observed between scroll length and the layer number of the precursor sheet. This trend can be interpreted through the relationship between Young’s modulus and crack formation. Nanoscrolls typically initiate at the edges of cracks that naturally form during plasma treatment, where tensile strain builds up due to lattice contraction during the WSe_2_-to-WSSe conversion. Monolayer WSSe exhibits a higher crack density owing to its low Young’s modulus and increased sensitivity to tensile strain. In contrast, stacking WSe_2_ and WSSe raises the overall Young’s modulus, enhancing resistance to strain and reducing crack formation. As a result, the scroll length increases with layer number due to a reduced density of initiation points. Thus, the nanoscroll length can be tuned by adjusting the number of layers in the TMD sheet during fabrication.

To further investigate scroll geometry across different thicknesses, the samples were examined by electron microscopy. Figure 3a shows HAADF-STEM images of nanoscrolls fabricated from trilayer WSSe/WSe_2_ sheets. In this sample, multiple nanoscrolls are observed forming in different directions and terminating upon collision within the same sheet. The sheet remains between adjacent scrolls, exhibiting alternating bright and dark contrast consistent with cracks introduced into the multilayer surface. An enlarged image reveals a multiwalled tubular structure composed of uniformly stacked TMD layers (Figure 3b). The measured interlayer spacing is 0.65 nm, in agreement with previously reported values for TMD nanoscrolls.^29,30^ Figure S3a-c presents TEM images of nanoscrolls formed from monolayer, bilayer, and trilayer sheets, respectively. The measured inner (outer) diameters are 7 (20) nm for monolayers, 70 (80) nm for bilayers, and 170 (220) nm for trilayers. These outer diameters are in good agreement with the AFM measurements. Notably, a strong correlation is observed between inner and outer diameters across all samples (Figure S3d–f).

**Figure 3:**
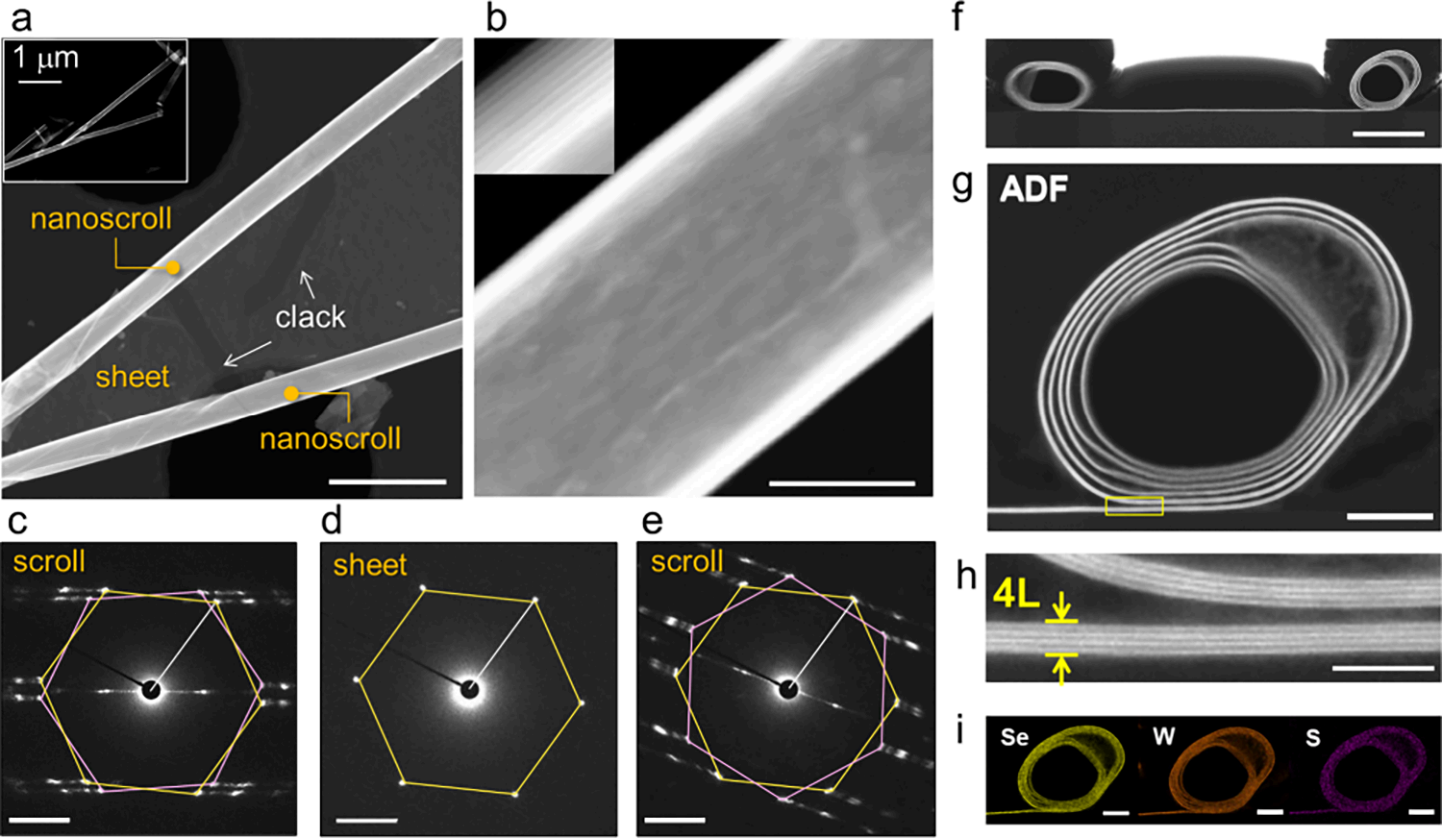
HAADF-STEM and EDX characterization of Janus WSSe/WSe_2_nanoscrolls. (a) Low-magnification HAADF-STEM image of nanoscrolls formed after solution treatment. The inset shows an overview image of the entire region containing the nanoscrolls. (b) Enlarged view of the left nanoscroll in (a). (c–e) Selected-area electron diffraction (SAED) patterns obtained from (c) the left nanoscroll, (d) the flat sheet, and (e) the right nanoscroll in (a). (f) Cross-sectional HAADF-STEM image of a nanoscroll composed of four-layer TMD sheets. (g) Enlarged view of the right side of the scroll in (f). (h) Further magnified image of the yellow boxed region in (g), showing the stacked layers. (i) Cross-sectional EDX elemental maps of Se, W, and S atoms corresponding to the nanoscroll in (g). Scale bars: (a) 500 nm; (b) 50 nm; (c–e) 2 nm^–^^1^; (f) 200 nm; (g) 50 nm; (h) 10 nm; (i) 50 nm.

Figure 3c-e shows the selected-area electron diffraction (SAED) patterns obtained from samples in Figure 3a. The selected-area diffraction pattern of the WSSe/WSe_2_ multilayer sheet displays a single set of 6-fold symmetric spots, characteristic of the hexagonal lattice of TMDs. This confirms that all stacked layers are aligned in the same crystallographic orientation. The lattice spacing of the (100) plane is estimated to be ∼ 0.27 nm, consistent with that of WSe_2_. In contrast, the diffraction pattern obtained from the nanoscroll exhibits two distinct sets of 6-fold symmetric spots, highlighted by pink and yellow lines. This is consistent with the expected pattern of a single-wall TMD nanotube with defined chirality.^45,46^ Both sets of diffraction spots indicate that the constituent layers within the nanoscroll share a common crystalline orientation. The hexagonal pattern marked in yellow shows better agreement with the sheet regions connected to the nanoscroll, suggesting that the pink and yellow patterns correspond to the front and back sides of the WSSe/WSe_2_ multilayer. The (100) lattice spacing in both regions was measured to be ∼ 0.27 nm, further supporting the structural consistency across the sheet and the scroll.

Cross-sectional HAADF-STEM imaging visualizes the internal structure of the nanoscrolls (Figure 3f,g). An enlarged view (Figure 3h) shows that the scroll is composed of four-layer TMD units. The inner cavity measures approximately 140 nm in width and 120 nm in height, forming a near-cylindrical cross-sectional geometry. The interlayer spacing appears slightly larger than the typical 0.65 nm, likely due to residual surface contaminants introduced during solution processing. Similar multiwalled scroll structures were observed in bilayer and trilayer regions (Figure S4). To investigate the elemental distribution, energy-dispersive X-ray spectroscopy (EDX) mapping was performed. Figure 3i shows cross-sectional EDX maps of S, Se, and W atoms in the nanoscroll corresponding to Figure 3g. The maps reveal spatially resolved elemental distributions across the layers, supporting the uniform substitution of sulfur derived from Janus WSSe.

Before the optical characterization, we have evaluated the photostability of Janus-based nanoscrolls and Janus WSSe flat sheet under ambient conditions. Figures S5 and S6 show the normalized Raman spectra and the A_1_ mode (288 cm^–1^) peak intensities of Janus WSSe samples before and after scrolling, measured at different irradiation times. Samples were exposed to 532 nm laser for 30 min. In monolayer sheets, the intensity dropped sharply within the first 5 min and plateaued at approximately 20% of the initial value. In contrast, the Raman intensities of 1L- and 8L-nanoscrolls remained unchanged after the same irradiation duration. This behavior, also observed in PL spectra (Figure S7), is attributed to photoinduced oxidation of WSSe.^47^ Moreover, both 1L- and 8L-nanoscrolls exhibited substantially enhanced stability compared to their unscrolled counterparts, suggesting that scrolling-induced self-encapsulation contributes to oxidation resistance under ambient conditions.

The anisotropic optical properties of the nanoscrolls were then investigated using polarized angle-resolved Raman spectroscopy. We measured three nanoscroll samples with diameters of approximately 30, 200, and 500 nm, corresponding to scrolls fabricated from monolayer WSSe, and two WSSe/WSe_2_ heterostructures with thicknesses of 5.2 and 8.4 nm, respectively. These thicknesses were determined by AFM (Figure S8), and the corresponding layer numbers of the multilayer regions were roughly estimated to be 8 and 12. The resulting 1L-, 8L-, and 12L-nanoscrolls exhibited heights (widths) of 30 nm (50 nm), 200 nm (400 nm), and 500 nm (1 μm), respectively (Figure S9). Figure 4a-c shows angle-resolved Raman spectra at polarization angles of 0°, 30°, 60°, and 90°. In these measurements, the polarization direction of the incident laser was varied by rotating the samples. The 1L-based nanoscroll exhibits Raman peaks at 280 cm^–^^1^ and 330 cm^–^^1^, corresponding to the A_1_ and E modes of Janus WSSe. The 8L- and 12L-based nanoscrolls show additional peaks at 250 cm^–^^1^, 260 cm^–^^1^, which are attributed to the A_1_′ and E′ modes of WSe_2_, respectively.

**Figure 4:**
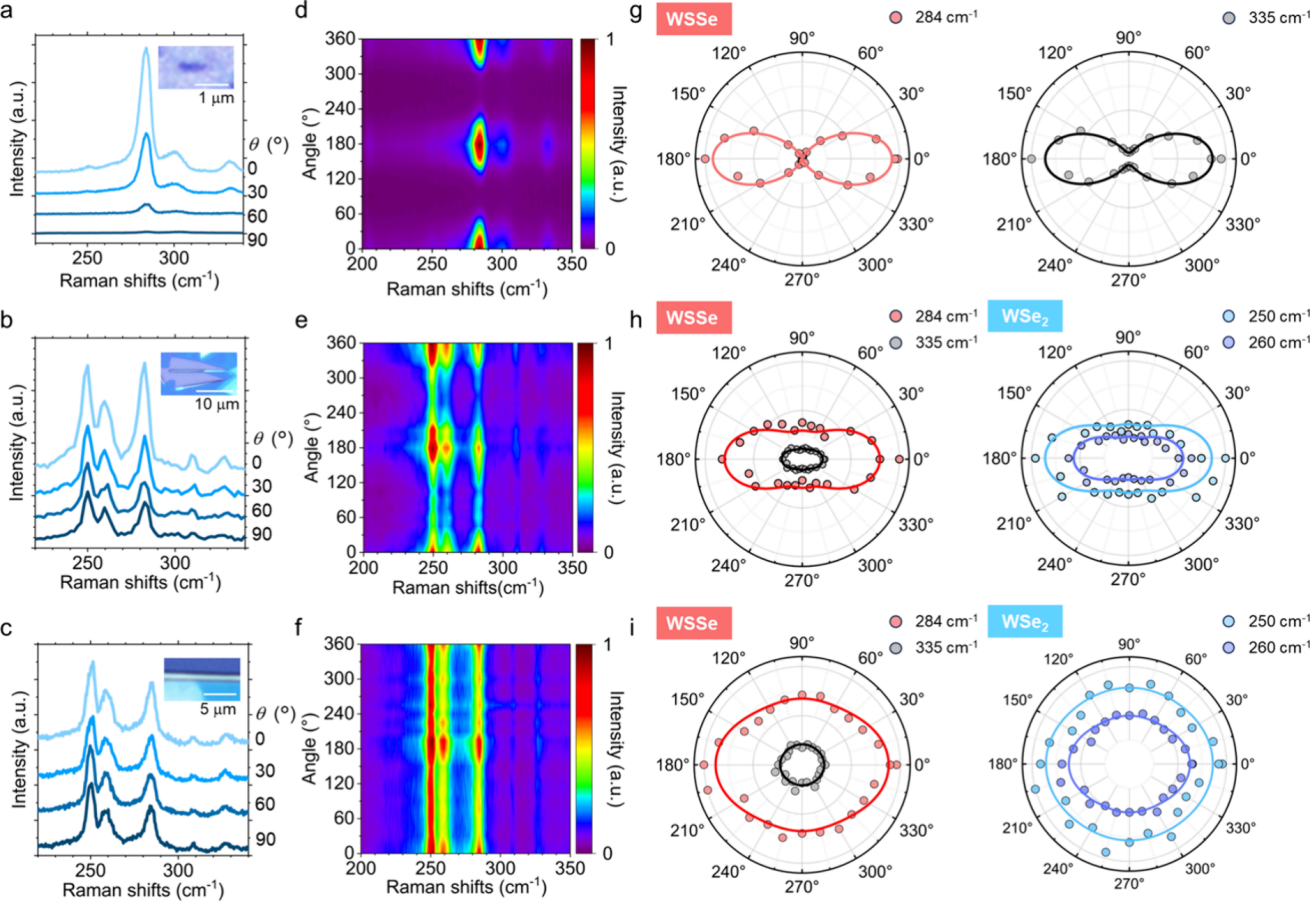
Angle-resolved polarized Raman spectroscopy of Janus WSSe/WSe_2_nanoscrolls. Raman spectra of nanoscrolls with diameters of (a) 30, (b) 200, and (c) 500 nm, measured at four representative polarization angles: 0, 30, 60, and 90°. (d–f) Polarized Raman intensity maps with diameters of (d) 30, (e) 200, and (f) 500 nm, shown as a function of polarization angle from 0 to 360°. Polar plots of the Raman peak intensities for nanoscrolls with diameters of (g) 30, (h) 200, and (i) 500 nm.

Figure 4d-f shows color maps of the Raman intensity measured on three different nanoscrolls. Figure 4g-i displays the polar plots of the A_1_ and E (or A_1_' and E') mode intensities for WSSe and WSe_2_. The 1L-based nanoscroll exhibits a clear 180° periodic variation in Raman intensity, with maxima at 0°, 180°, and 360°, where the polarization is aligned with the scroll axis. This trend is consistent with that of monolayer MoSSe nanoscrolls reported in our previous work.^38^ The 4L-based scrolls show similar angular dependence, although the reduction in intensity under perpendicular polarization is less pronounced. In contrast, the Raman intensity of 12L-based scrolls shows almost no dependence on polarization. These results demonstrate that the polarization-dependent Raman response is strongly correlated with scroll diameter. The anisotropic Raman response can be partly attributed to the anisotropic optical absorption of nanoscrolls, in particular the weak absorption of photons with out-of-plane polarization.^48^ In addition, the depolarization effect^49^ also plays an important role: for narrow scrolls with widths much smaller than the laser wavelength, the incident electric field is effectively screened. For wider scrolls (e.g., > 1 μm), the screening becomes negligible, and the behavior resembles that of planar sheets. This size-dependent optical response highlights the utility of nanoscrolls as a platform for exploring nanoscale optical phenomena, including depolarization effects.

To characterize the Janus-based nanoscroll structures, we conducted second-harmonic generation (SHG) measurements. To evaluate the relative intensity of SHG in nanoscroll structures, we first obtained spatial images of the SHG signal. Figure 5a–c shows an optical microscope image of the nanoscrolls, the corresponding AFM image, and the integrated SHG intensity map. The bright contrast at the center of the optical and AFM images corresponds to nanoscrolls formed from multilayer WSSe/WSe_2_ sheets. In Figure S10, the AFM image reveal step-like features with heights of approximately 8–11 nm. Even at the thinnest regions (∼8 nm), the thickness corresponds to at least 11 layers, assuming an average interlayer spacing of ∼ 0.7 nm.

**Figure 5:**
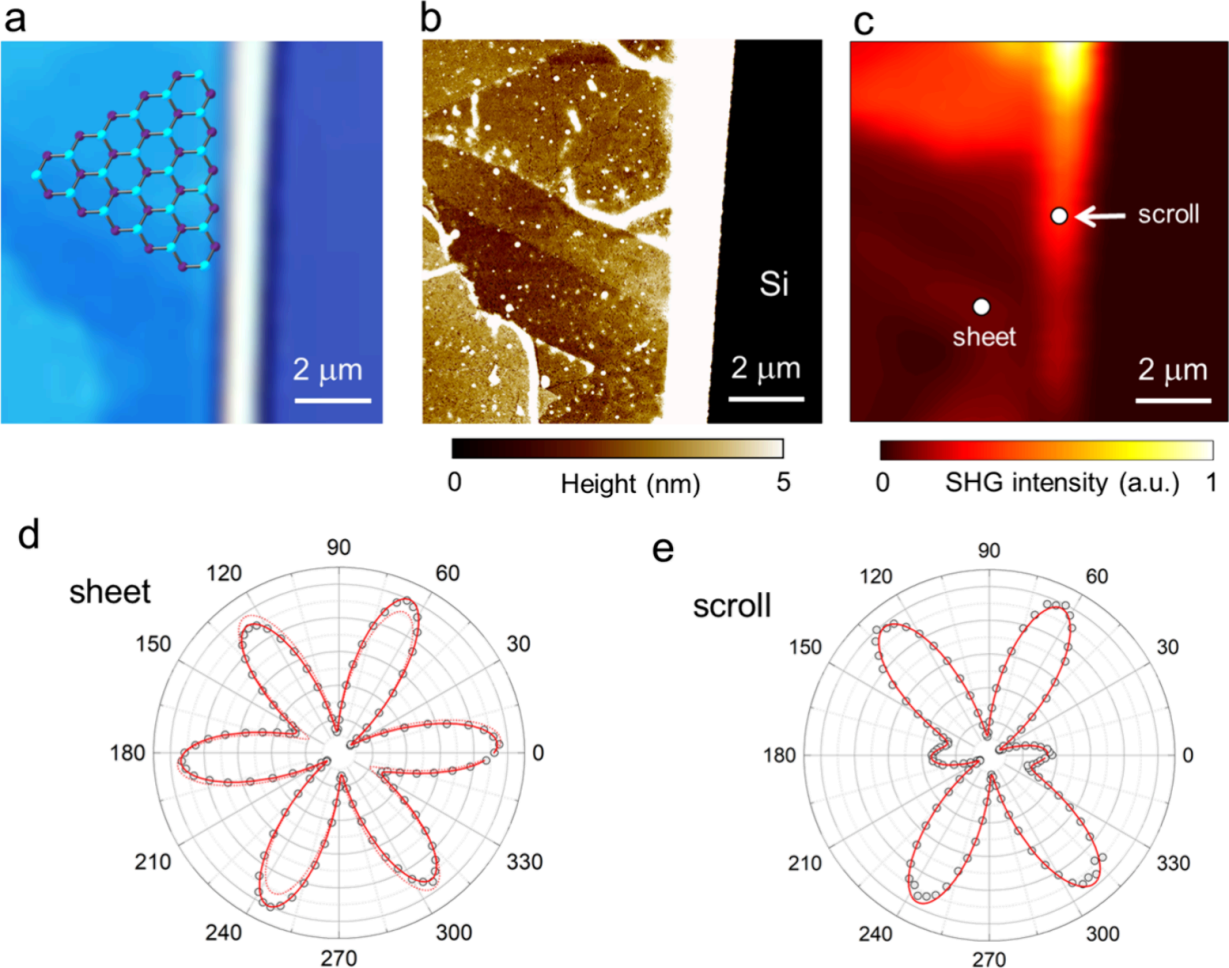
SHG characterization of Janus WSSe/WSe_2_nanoscroll. (a) Optical microscope image, (b) corresponding AFM topography image, and (c) SHG intensity map of the same region showing a nanoscroll and a flat sheet. (d, e) Polar plots of SHG intensity as a function of polarization angle *θ*, measured at (d) WSSe/WSe_2_ heterostructure sheet and (e) nanoscroll, corresponding to the white circles in (c). Black circles are the experimental data. Red solid curves are fits using the strain-inclusive model. For (d), a dashed curve shows the reference fit obtained without the strain effect, for comparison. Intensities are normalized to the maximum in each panel.

In TMDs, SHG activity is known to depend on the layer number due to inversion symmetry: odd-layered structures lack inversion symmetry and thus exhibit strong SHG, while even-layered ones possess inversion symmetry, leading to the cancellation of SHG signals.^50^ Accordingly, we observed significant SHG intensity from odd-layer regions and suppressed signals from even-layer regions. Notably, the SHG intensity from nanoscrolls was enhanced by a factor of 2.6 compared to that from the corresponding flat sheets. This enhancement contrasts with a previous study on few-layer MoS_2_, which showed a monotonic decrease in SHG intensity with increasing layer number due to light absorption and interlayer coupling.^50^ STEM imaging suggests that the present nanoscrolls exhibit weak interlayer coupling due to nanoscale gaps between adjacent layers. This weak coupling may prevent the electronic structure modification and allow for the constructive overlap of SHG signals from multiple layers.

Figure 5d presents a polar plot of SHG intensity measured in a copolarized configuration from the WSSe/WSe_2_ sheet (white circle in Figure 5c). The SHG response exhibits a characteristic 6-fold symmetry, consistent with the hexagonal lattice symmetry of TMDs. In such systems, the SHG intensity reaches its maximum along the armchair direction, where mirror symmetry is broken. Based on this, we assigned the 0° direction to the armchair axis and 30° to the zigzag axis.

Figure 5e shows a polar plot of the SHG intensity obtained from the nanoscroll at the position indicated by the white circle in Figure 5c. The scroll exhibits local intensity maxima at the same angular directions, indicating that the scroll retains the crystalline orientation of the original WSSe/WSe_2_ sheet. The nanoscroll axis is oriented nearly parallel to zigzag direction, with a chiral angle estimated to be around 27°. Interestingly, the SHG intensity decreases near 0° and 180°, i.e., along the direction perpendicular to the nanoscroll axis. This behavior can be reproduced by a strain-inclusive SHG model,^51^ without invoking anisotropic effective electric fields inside the scroll (Figure S11).^34^ The fitting procedure is provided in the Supporting Information. The estimated principal strain axis (∼84°) aligns with the nanoscroll axis (∼88°), underscoring that the scrolling geometry introduces anisotropic strain and thereby modulates the second-order nonlinear optical response.

Such anisotropic strain is considered a characteristic feature of multilayer nanoscrolls. For monolayers, theoretical calculations suggest that the optimal diameter is around 10 nm, and the previous experiments frequently observe inner diameters in the range of 5–10 nm.^38,39^ These results indicate that the most stable structures form in the innermost core layer of the scroll during strain relaxation. However, since the outer layers of a monolayer scroll exceed 10 nm in diameter, complete strain relaxation is not possible. Similarly, the larger diameter of the present multilayer nanoscrolls also prevents full relaxation of the Janus WSSe layer. Meanwhile, bending induces strain in the WSe_2_ layers. Thus, multilayer nanoscrolls inherently stabilize as strained structures, due to van der Waals interactions between the Janus and non-Janus layers. In this sense, they provide a platform where strain can be effectively tuned by varying the number of layers.

Finally, we note key differences between Janus nanoscrolls and conventional planar Janus monolayers or vertical heterostructures. First, their nanotube-like geometry gives them chirality and quasi-one-dimensionality, enabling unique physical phenomena such as bulk photovoltaic effects and anisotropic optical responses. Second, nanoscrolls possess a scroll-specific self-overlapping configuration, in which the overlapping regions originate from the same sheet. This geometry gives rise to intralayer interactions that can modulate the electronic structure, including band bending and type-II band alignment driven by the built-in dipole, as a previous theoretical study.^52^ Furthermore, depending on the scrolling angle, they can form one-dimensional twisted bilayer or multilayer moiré structures within the same sheet. Exploring these possibilities is an exciting direction for future research.

## Conclusions

We fabricated nanoscrolls from multilayer WSe_2_ incorporating a Janus WSSe monolayer by the solution-assisted roll-up process. The resulting nanoscroll structures were characterized by AFM and HAADF-STEM, and their elemental composition was confirmed by EDX mapping. Raman/PL spectroscopy demonstrated that the WSSe nanoscroll structures exhibit high photostability under ambient conditions. Polarized angle-resolved Raman spectroscopy revealed anisotropic optical responses, while polarization-resolved SHG probed the nonlinear optical behavior arising from structural symmetry. Notably, we found that the inclusion of a Janus WSSe monolayer facilitates the scrolling of multilayer TMD sheets, and that the resulting scroll diameter and length can be tuned by varying the number of precursor layers. These findings establish Janus-based nanoscrolls as a versatile platform for exploring chirality-, diameter-, and polarization-dependent properties in low-dimensional systems, while providing design principles for strain-engineered TMD nanostructures. Such a fundamental framework also opens opportunities for nanoscrolls to serve as tunable model systems for studying nonlinear optical responses and high-surface-area systems for sensing, catalysis, and energy storage.

## Experimental Methods

### Sample Preparation

Monolayer and multilayer WSe_2_ was synthesized on SiO_2_ (285 nm)/Si substrates by using a lab-built CVD system.^53^ WO_3_ and selenium powder were used as precursors, and KBr was employed as a growth promoter. WO_3_ (50–90 mg), KBr (30–50 mg), and Se (2 g) powders were placed at positions 1, 3, and 27 cm upstream from the substrate inside a quartz tube, respectively. A mixture of H_2_/N_2_ gas (H_2_ = 0.7%) was continuously flowed through the quartz tube at a rate of 450 sccm. During growth, the substrates and Se source were heated to 950 and 350 °C, respectively. Janus WSSe was prepared by plasma-assisted surface atom substitution of WSe_2_. The CVD-grown WSe_2_ samples were placed in a quartz tube wrapped with a copper coil, and sulfur powder was placed upstream in the quartz tube. The input power and reaction time were set to 15–30 W and 30–60 min, respectively. For nanoscroll formation, a PMMA/chloroform solution was spin-coated on the substrate to facilitate the partial detachment and spontaneous scrolling of Janus WSSe monolayer and WSSe/WSe_2_ heterostructures.

### Transfer Process

To observe the nanoscrolls by TEM/STEM, the samples were transferred onto a carbon-coated TEM grid. To prepare the support films, a PMMA/chloroform solution was spin-coated onto the substrates. The substrate was then immersed in an etching solution (1 mol/L KOH) at 80 °C for several hours. The PMMA film was peeled off from the substrate, rinsed with pure water, and subsequently transferred onto a TEM grid. After drying, the PMMA film was dissolved using acetone.

### Characterizations

Raman and PL spectra were measured under 532 nm excitation in a backscattering configuration using a microspectrometer (inVia, Renishaw). For polarization-resolved Raman spectroscopy measurements, the angle between the nanoscroll axis and the incident laser polarization was controlled by rotating the samples. Topographic images were acquired using an AFM (NX10, Park Systems). Samples for cross-sectional TEM observation were prepared using the focused ion beam method. For cross-sectional observation, HAADF images and EDX maps were acquired using a high-resolution electron microscope (HD-2700, Hitachi High-Technologies) equipped with a silicon drift detector (Ultim Max TLE, Oxford Instruments).

For the SHG measurements, a femtosecond pulse laser with a center wavelength of 1560 nm was used as a pump source, where the repetition rate and the pulse width were 75.4 MHz and ∼ 250 fs, respectively. The pump beam was focused with a 50x objective lens and a spot size on the sample plane was ∼ 2 μm. Average pump power was kept less than 10 mW to avoid damaging the sample. A half-waveplate was used to control the polarization angle θ, and a polarizer was inserted in front of a single-photon avalanche diode to detect the second-harmonic signals with polarization parallel to that of the pump beam. A dichroic mirror and a long-pass filter were also used to extract the second-harmonic light at 780 nm.

## Supplementary Material



## Notes

Evaluation of strain energy and the stable diameter as a function of layer number; analysis of linear polarization dependence of the SHG intensity; optical images of as-prepared WSSe/WSe_2_ sheets and nanoscrolls; strain energy and the stable diameter at different layer numbers; TEM characterization of nanoscrolls; cross-sectional HAADF-STEM characterization of nanoscrolls; time-dependent Raman spectra of Janus WSSe samples under ambient conditions; time-dependent Raman peak intensity plots of Janus WSSe samples under ambient conditions; time-dependent PL characterization of Janus WSSe samples under ambient conditions; topography and height profiles of Janus WSSe/WSe_2_ heterostructures; topography of nanoscrolls formed from different layer numbers; height profiles of a Janus WSSe/WSe_2_ heterostructure sheet and a nanoscroll; and polarization dependence of SHG intensity (PDF)
