# Supplementary material for: Tunable-Diameter Nanoscrolls from Janus WSSe/WSe2 Heterostructures
Source: ACS Nano. 2025 Sep 23;19(39):34918–27. doi: 10.1021/acsnano.5c10877 (PMC12509307; doi:10.1021/acsnano.5c10877)
Supplement: Supplementary file 1 [file nn5c10877_si_001.pdf]

Supporting Information for

Tunable-Diameter Nanoscrolls from Janus WSSe/WSe$_2$ Heterostructures

Masahiko Kaneda[1,2], Wenjin Zhang[1], Dingkun Bi[3,4], Tianyishan Sun[3,4], Hiroto Ogura[3,4], Takahiko Endo[1,2], Yuta Takahashi[5], Shun Fujii[5], Toshiaki Kato[3,4], and Yasumitsu Miyata[1,2]**

1. Research Center for Materials Nanoarchitectonics (MANA), National Institute for Materials Science (NIMS), Tsukuba 305-0044, Japan
2. Department of Physics, Tokyo Metropolitan University, Hachioji 192-0397, Japan.
3. Graduate School of Engineering, Tohoku University, Sendai, 980-8579, Japan
4. Advanced Institute for Materials Research (AIMR), Tohoku University, Sendai, 980-8577, Japan
5. Department of Physics, Faculty of Science and Technology, Keio University, Yokohama, 223-8522, Japan

Address correspondence to: miyata.yasumitsu@nims.go.jp; kato12@tohoku.ac.jp

Evaluation of strain energy and the stable diameter as a function of layer number

The strain energy density per unit area U of a non-Janus monolayer transition metal dichalcogenide (TMD) can be expressed as

$$U = \frac{1}{2} D\kappa^2,$$

where D is the bending rigidity of monolayer TMD, and κ is the curvature.[1-3] The curvature is defined as the inverse of the radius, and in terms of the diameter d, it can be written as $\kappa = 2/d$. For an N-layer multilayer TMD with thickness t, the effective bending rigidity D_{eff} can be expressed as

$$D_{\mathrm{eff}} = f(N) \frac{Et^3}{12(1-\nu^2)},$$

where E is the Young's modulus, ν is the Poisson's ratio, and $f(N)$ is a function of the number of layers N, typically ranging from 0.4 to 0.7 for MoS_2.[4] Here, for simplicity, we consider the limiting case of perfectly bonded layers within a thin-plate bending model, and treat E, ν, and $f(N)$ as constants independent of N. Under this approximation, D_{eff} scales with the cube of t (i.e., with the cube of N) and is equal to the monolayer bending rigidity D multiplied by this term,

$$D_{\mathrm{eff}} = N^3 D.$$

For monolayer WSSe, the strain energy density can be expressed as

$$U = \frac{1}{2} D(\kappa - \kappa_0)^2 + \text{constant term},$$

where κ_0 is the spontaneous curvature of monolayer Janus WSSe, and we assume that D is the same for monolayer WSSe and WSe_2 for simplicity. For an N-layer WSSe/WSe_2 heterostructure consisting of a monolayer Janus WSSe on top and $N-1$ layers of WSe_2, the strain energy density can be written as

$$U = \frac{1}{2} D_{\mathrm{eff}}(\kappa - \kappa_{\mathrm{eff}})^2 + \text{constant term},$$

where κ_{eff} is the effective spontaneous curvature of the N-layer heterostructure. From the balance of bending moments,

$$\kappa_{\mathrm{eff}} D_{\mathrm{eff}} = \kappa_0 D,$$

which leads to

$$\kappa_{\mathrm{eff}} = \frac{\kappa_0}{N^3}.$$

The value of κ_0 is estimated to be around 0.2 nm^{-1} (corresponding to a diameter of 10 nm) based on first-principles calculations.[5] Thus, the effective equilibrium diameter (N-layer) d_{eff} can be expressed as

$$d_{\text{eff}} = \frac{2}{\kappa_{\text{eff}}} = \frac{2N^3}{\kappa_0} = 10N^3 \text{ (nm)}.$$

In contrast, in the limiting case of independently sliding layers,

$$D_{\text{eff}} = ND,$$

which leads to

$$\kappa_{\text{eff}} = \frac{\kappa_0}{N},$$

$$d_{\text{eff}} = 10N \text{ (nm)}.$$

This yields a smaller d_{eff} than in the perfectly bonded case.

Figure S2 shows the calculated strain energy density versus diameter, as well as the most stable diameter for a monolayer (*N*=1) and heterostructures (*N*=2~4). In both cases, the strain energy density increases rapidly below d_{eff}, limiting the minimum inner diameter formed in the scroll (Figure S2a,b). In Figure S2c, the experimental inner diameters lie between these two limits. This trend can be attributed to partial interlayer sliding in nanoscrolls, as reported in bending experiments of multilayer MoS_2.[4] A more precise statistical evaluation of the inner diameter and a theoretical assessment of the bending rigidity are left for future work.

Analysis of linear polarization dependence of the SHG intensity

We analyzed the polarization-dependent SHG intensities of the sheet and scroll using an SHG model incorporating strain effect.[6] This model accounts for the modification of nonlinear susceptibility under uniaxial strain, and the SHG intensity can be expressed as follows:

$$I_{\parallel}^{(2)}(2\omega) \propto (A\cos(3\phi) + B\cos(2\theta_{\text{S}} + \phi))^2,$$

where

$$A = (1-\nu)(p_1 + p_2)(\varepsilon_{\text{xx}} + \varepsilon_{\text{yy}}) + 2\chi_0$$
$$B = (1+\nu)(p_1 - p_2)(\varepsilon_{\text{xx}} - \varepsilon_{\text{yy}}).$$

Here, p_1 and p_2 are the photoelastic parameters, ε_{xx} and ε_{yy} are the principal strains, θ_S is the principal strain orientation, ϕ the polarization angle, and χ_0 is the nonlinear susceptibility parameter of the unstrained crystal lattice. To reproduce the experimental results, we use the following expression for the background with weak polarization dependence:

$$I_0 + I'\cos(2(\phi - \varphi_0)),$$

where I_0, I', and ϕ_0 are parameters probably originating from the polarization dependence of the optical system. This background has negligible influence on the main results (Figure S11a,b). The background amplitude and phase are nearly identical for the sheet and the scroll, suggesting that the twofold-symmetric component originating from the optical system is common to both samples. These expressions reproduce well the angular dependence of the SHG intensity observed in both the sheet

and the scroll.

Next, we examined the influence of the polarization dependence of the effective electric field. In nanoscrolls, the effective electric field intensity may decrease for polarization perpendicular to the scroll axis, depending on the diameter and geometry.[7] To evaluate this effect, we performed a fit using the equation without including strain, by applying a correction factor of $\{E_0 + E' \, cos(\phi - \theta_{\mathrm{E}})\}^4$:

$$I_{\parallel}^{(2)}(2\omega) \propto A\cos^2(3\phi)\{E_0 + E' \, cos(\phi - \theta_{\mathrm{E}})\}^4$$

Here, θ_{E} is the angle at which the electric field reaches its minimum. As shown in Figure S11c, this model fails to reproduce the main peak positions, and the obtained θ_{E} ~170° deviates from the perpendicular direction (~178°). Given that the present scrolls have a width exceeding 1 μm, the polarization dependence of the effective electric field is considered negligible. These results support the conclusion that the primary origin of the observed SHG anisotropy is axial strain induced by scrolling.

The strain magnitude and orientation, represented by *B*/*A* and θ_{S}, are estimated to be ~0.1 and 55° for the sheet, and ~0.47 and 84° for the scroll, respectively. The small anisotropy observed in the sheet is probably due to thermal shrinking of the substrate after growth, as well as changes in lattice constant induced by atom substitution process. For the scroll, the value of θ_{S} (~84°) strongly suggests alignment with the scroll axis (~88∘). The increase in *B*/*A* is likely due to scrolling-induced uniaxial or shear strain.

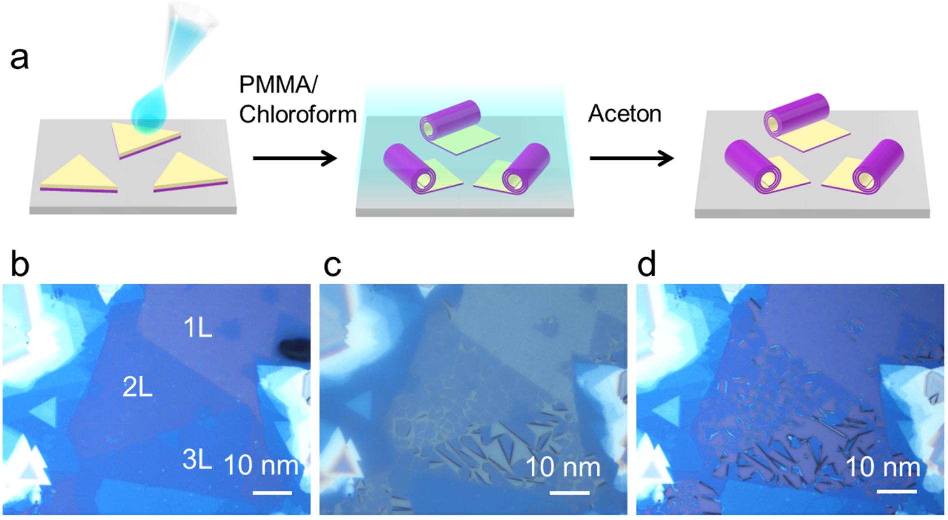

Figure S1. Optical images of as-prepared WSSe/WSe$_2$ sheets and nanoscrolls. (a) Schematic illustration of the solution treatment. Optical microscope images taken (b) before and (c) after spin-coating with PMMA/chloroform solution, and (d) after washing with acetone.

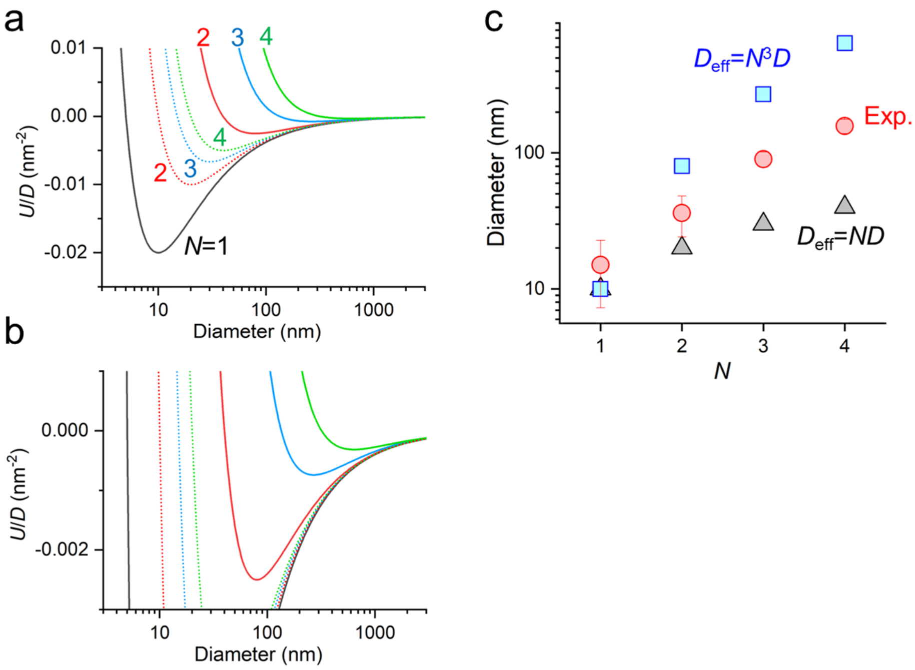

Figure S2. Strain energy and the stable diameter at different layer numbers. (a,b) Strain energy density normalized by the monolayer bending rigidity, U/D, plotted as a function of diameter, d, for monolayer WSSe (N=1) and WSSe/WSe$_2$ heterostructures (N=2~4) in the two limiting cases: (solid line) perfectly bonded multilayer ($D_{\mathrm{eff}} \propto N^3$) and (dashed line) independently sliding layers ($D_{\mathrm{eff}} \propto N$). For each N, the energy of a flat sheet is set to zero. (c) Comparison of the estimated average inner diameters and the effective equilibrium diameter d_{eff} for N =1–4. In this work, the average inner diameters (red circle) were roughly estimated from the height distributions in AFM results (Figure 2) using the relationship between inner and outer diameters from TEM observations (Figure S3f). d_{eff} was calculated in the two limiting cases: (blue square) perfectly bonded multilayer ($D_{\mathrm{eff}}=N^3D$) and (black triangle) independently sliding layers ($D_{\mathrm{eff}}=ND$).

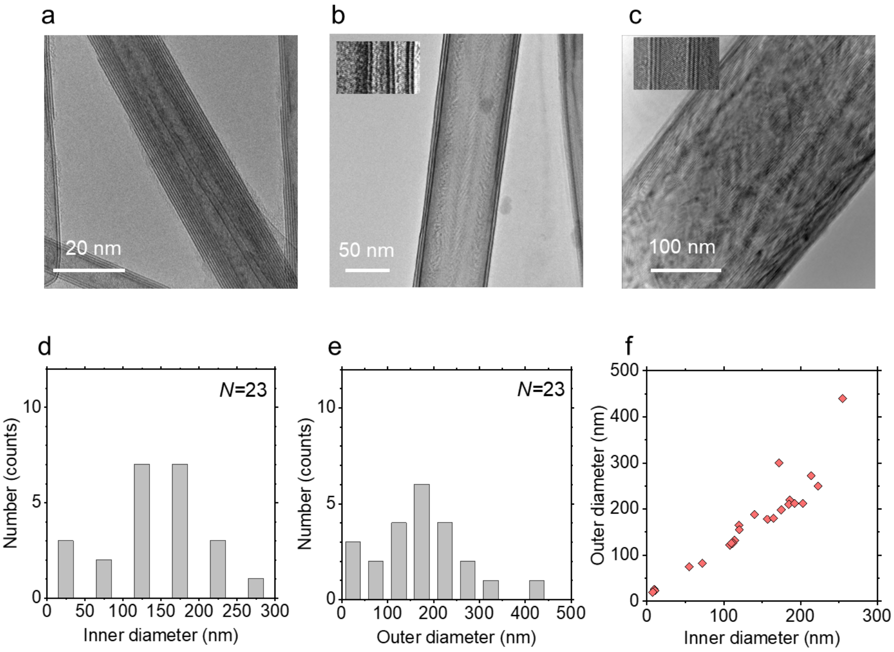

Figure S3. TEM characterization of nanoscrolls. TEM images of nanoscrolls formed from (a) monolayer WSSe, (b) bilayer, and (c) trilayer WSSe/WSe_2. Histogram of (d) inner diameter and (e) outer diameter of nanoscrolls. These data were obtained from nanoscrolls formed from regions with different layer numbers. (f) A plot of inner diameter versus outer diameter of the Janus WSSe/WSe_2 nanoscrolls. A positive correlation between the inner and outer diameters is observed.

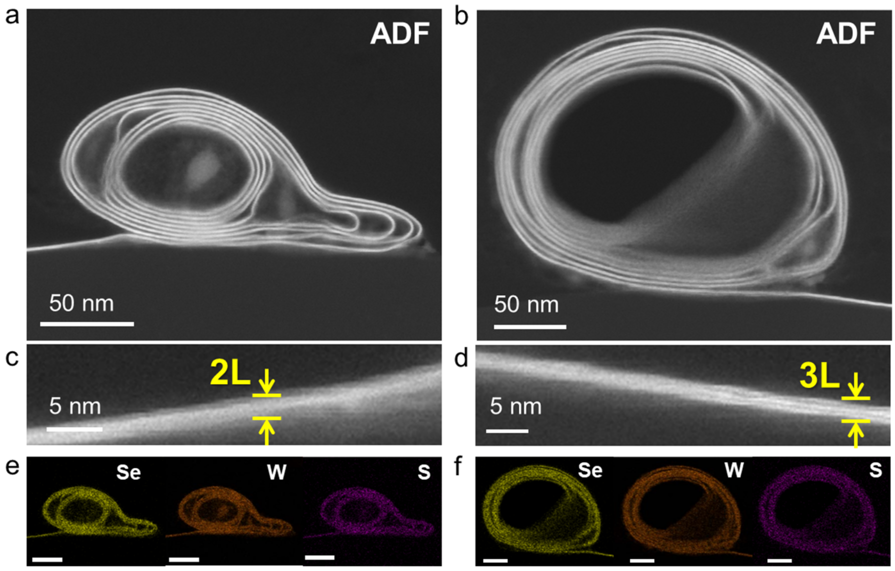

Figure S4. Cross-sectional HAADF-STEM characterization of nanoscrolls. (a,b) Cross-sectional HAADF-STEM images of nanoscrolls formed from WSSe/WSe_2 heterostructure sheets. (c,d) Enlarged views of the lower sheet regions in (a) and (b), respectively. (e,f) EDX elemental maps corresponding to (a) and (b), respectively, showing the spatial distributions of Se, W, and S atoms. Scale bars are 50 nm in (e) and (f).

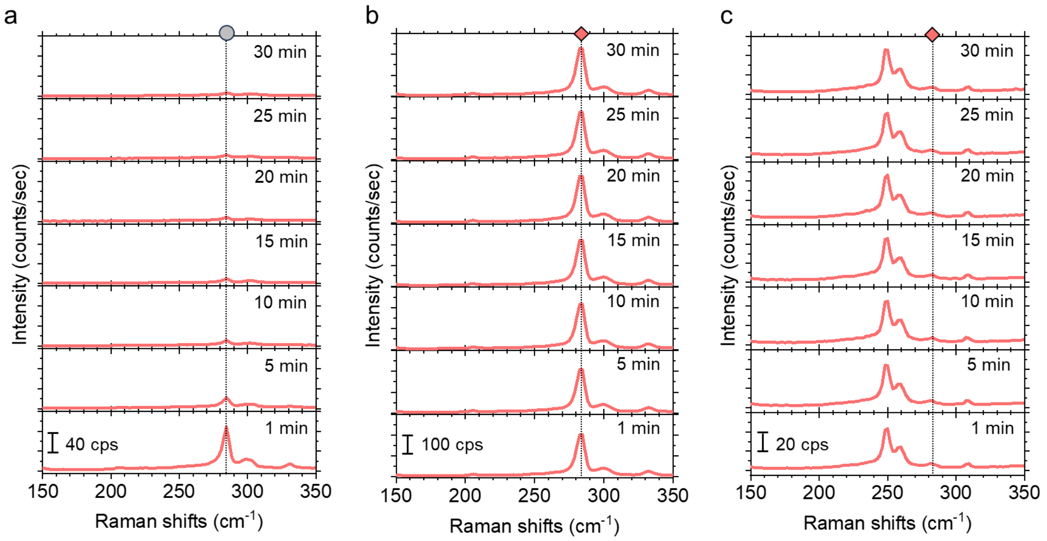

Figure S5. Time-dependent Raman spectra of Janus WSSe samples under ambient conditions. Raman spectra of (a) a monolayer WSSe flat sheet, (b) a 1L-nanoscroll, and (c) an 8L-nanoscroll (WSSe/WSe_2) measured at different irradiation times under ambient conditions. All measurements were performed using a 532 nm excitation laser (70 μW) and a 100× objective lens with a numerical aperture (NA) of 0.85.

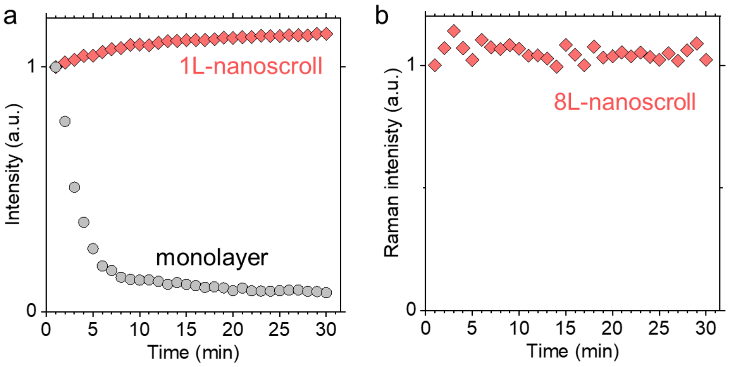

Figure S6. Time-dependent Raman peak intensity plots of Janus WSSe samples under ambient conditions. Raman peak intensity of the A_1 mode (288 cm^{-1}) plotted as a function of irradiation time for (a) a WSSe monolayer and a 1L-nanoscroll, and (b) an 8L-nanoscroll (WSSe/WSe_2). All measurements were performed using a 532 nm excitation laser (70 μW) and a 100× objective lens with a numerical aperture (NA) of 0.85.

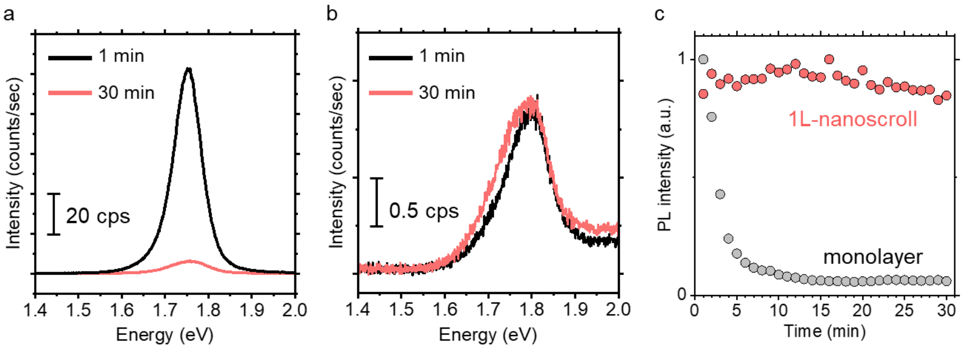

Figure S7. Time-dependent PL characterization of Janus WSSe samples under ambient conditions. PL spectra of (a) a Janus WSSe monolayer and (b) a 1L-nanoscroll measured at different irradiation times. (c) PL intensity of the A exciton (1.8 eV) plotted as a function of irradiation time for the WSSe monolayer and the 1L-nanoscrolls. All measurements were performed using a 532 nm excitation laser (70 µW) and a 100× objective lens with a numerical aperture (NA) of 0.85.

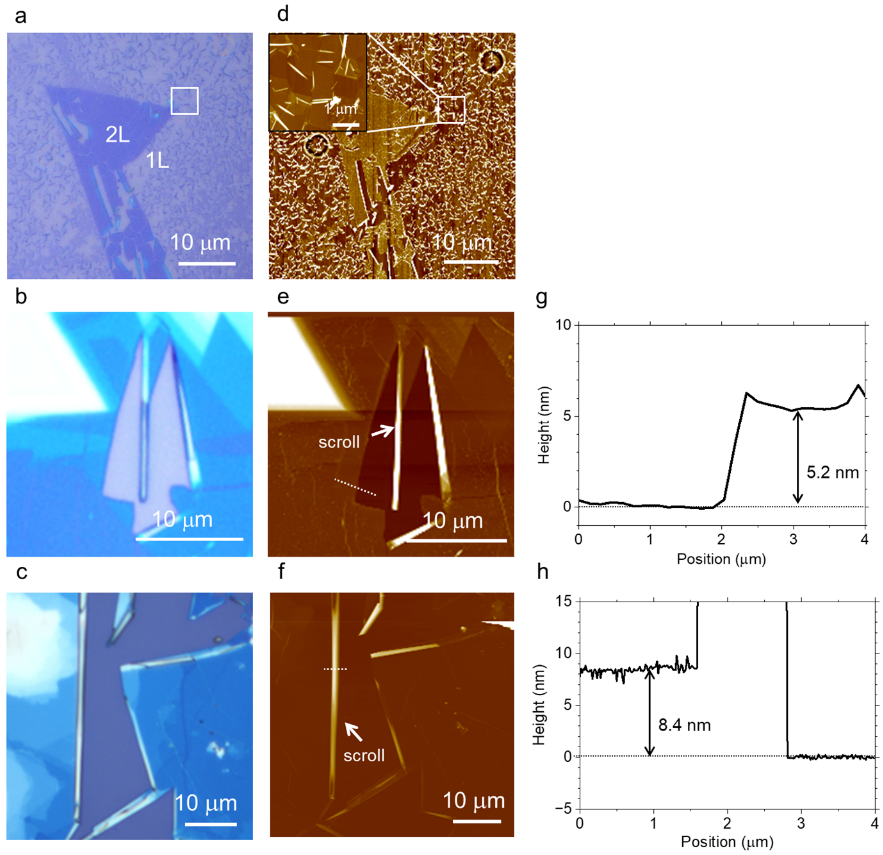

Figure S8. Topography and height profiles of Janus WSSe/WSe_2 heterostructures. Optical microscope images of WSSe-based nanoscrolls formed from (a) monolayer, (b) 8-layer, and (c) 12-layer sheets. (d-f) AFM images corresponding to (a-c), respectively. (g,h) Height profiles taken along the white lines in (e) and (f).

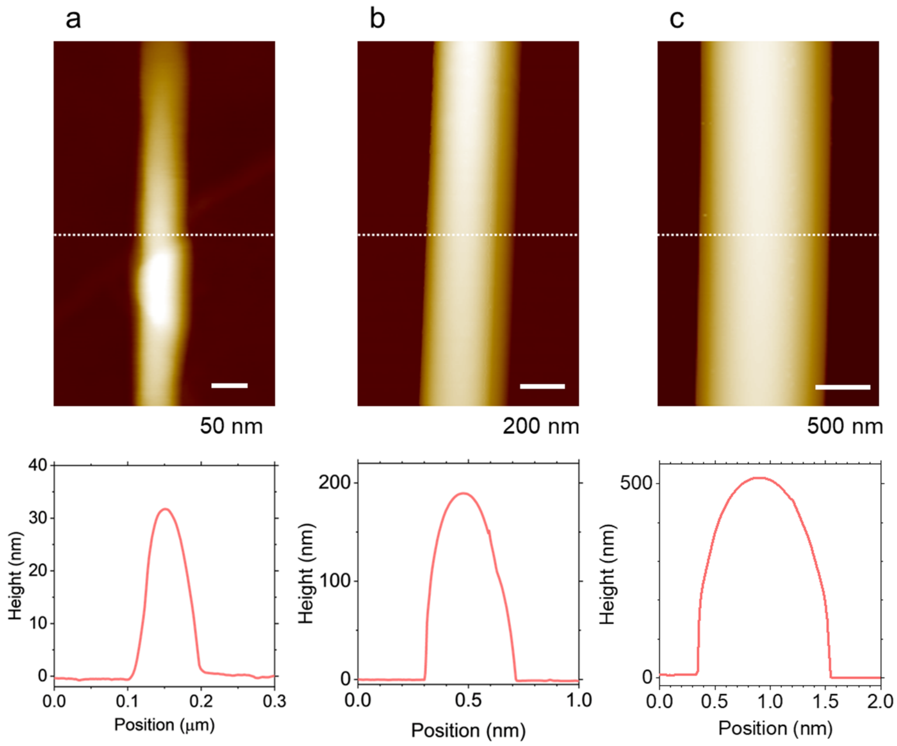

Figure S9. Topography of nanoscrolls formed from different layer numbers. AFM images and corresponding height profiles of nanoscrolls formed from (a) 1L WSSe, (b) 8L and (c) 12L Janus WSSe/WSe_2 sheets. The height profiles were obtained along the white dotted lines shown in the AFM images.

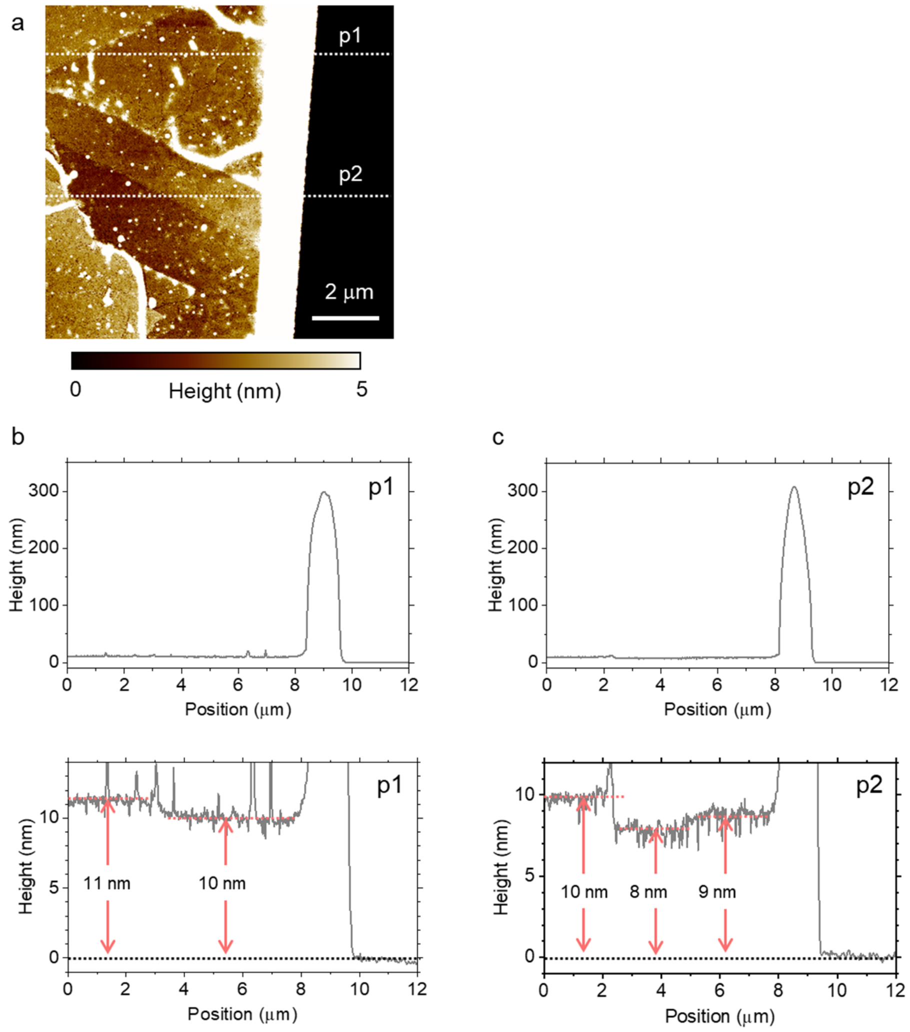

Figure S10. Height profiles of a Janus WSSe/WSe_2 heterostructure sheet and a nanoscroll. (a) AFM image of a Janus WSSe/WSe_2 sheet and a nanoscroll, (b,c) Height profiles along the white dotted lines labeled p1 and p2 in (a), respectively. The lower panels in (b, c) show magnified height profiles in the flat sheet regions, indicating the measured thicknesses of individual steps.

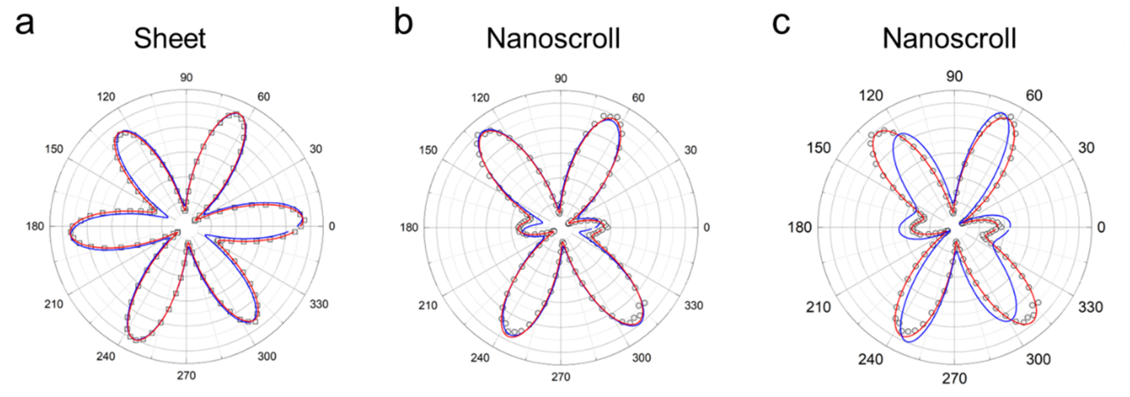

Figure S11. Polarization dependence of SHG intensity. (a-c) Polar plots of SHG intensity as a function of polarization angle θ, measured for (a) the flat sheet and (b,c) the nanoscroll of WSSe/WSe_2 heterostructures. The black circles correspond to the experimental data in Figure 5d,e. In (a) and (b), the solid curves show the fitting results using the strain-inclusive model with an anisotropic background (red) and with a constant background (blue). In (c), the solid curves show the fitting results using the strain-inclusive model (red) and the anisotropic effective electric field model (blue) with an anisotropic background. All intensities are normalized to the maximum in each panel.

(1) Seifert, G.; Terrones, H.; Terrones, M.; Jungnickel, G.; Frauenheim, T. Structure and Electronic Properties of MoS_2 Nanotubes. *Phys. Rev. Lett.* **2000**, *85*, 146-149.

(2) Jiang, J.-W.; Qi, Z.; Park, H. S.; Rabczuk, T. Elastic Bending Modulus of Single-Layer Molybdenum Disulfide (MoS_2): Finite Thickness Effect. *Nanotechnology* **2013**, *24*, 435705.

(3) Xiong, S.; Cao, G. Bending Response of Single Layer MoS_2. *Nanotechnology* **2016**, *27*, 105701.

(4) Wang, G.; Dai, Z.; Xiao, J.; Feng, S.; Weng, C.; Liu, L.; Xu, Z.; Huang, R.; Zhang, Z. Bending of Multilayer Van Der Waals Materials. *Phys. Rev. Lett.* **2019**, *123*, 116101.

(5) Gao, Y.; Kaneda, M.; Endo, T.; Nakajo, H.; Aoki, S.; Kato, T.; Miyata, Y.; Okada, S. Strain-Induced Scrolling of Janus Wsse. *Phys. Rev. B* **2024**, *110*, 035414.

(6) Mennel, L.; Furchi, M. M.; Wachter, S.; Paur, M.; Polyushkin, D. K.; Mueller, T. Optical Imaging of Strain in Two-Dimensional Crystals. *Nat. Commun.* **2018**, *9*, 516.

(7) Qian, Q.; Zu, R.; Ji, Q.; Jung, G. S.; Zhang, K.; Zhang, Y.; Buehler, M. J.; Kong, J.; Gopalan, V.; Huang, S. Chirality-Dependent Second Harmonic Generation of MoS_2 Nanoscroll with Enhanced Efficiency. *ACS Nano* **2020**, *14*, 13333-13342.
